# Complications of Pregnancy and the Risk of Developing Endometrial or Ovarian Cancer: A Case-Control Study

**DOI:** 10.3389/fendo.2021.642928

**Published:** 2021-04-30

**Authors:** Yang Liu, Xingyu Chen, Jiayi Sheng, Xinyi Sun, George Qiaoqi Chen, Min Zhao, Qi Chen

**Affiliations:** ^1^ School of Medicine, Nanjing Medical University, Nanjing, China; ^2^ Department of Obstetrics & Gynaecology, The University of Auckland, Auckland, New Zealand; ^3^ School of Medicine, The University of Manchester, North Manchester, United Kingdom; ^4^ Department of Gynaecology, The Affiliated Wuxi Maternity and Child Health Care Hospital of Nanjing Medical University, Wuxi, China

**Keywords:** preeclampsia, GDM, IUGR, endometrial cancer, ovarian cancer

## Abstract

**Background:**

The association of complications of pregnancy and the risk of developing gynecological cancer is controversial with the limited study. In this study, we investigated the association of preeclampsia, or gestational diabetes mellitus (GDM), or large for gestational age (LGA), or intrauterine growth restriction (IUGR) and the risk of endometrial or ovarian cancer.

**Methods:**

In this case-control study, 189 women with endometrial cancer and 119 women with ovarian cancer were included. 342 women without gynecological cancers were randomly selected as a control group. Data on the history of pregnancy and age at diagnosis of gynecological cancer as well as the use of intrauterine devices (IUDs) were collected.

**Results:**

Women with a history of preeclampsia or IUGR did not have an increased risk of developing endometrial or ovarian cancer. While women with a history of GDM or with the delivery of LGA infant increased the risk of developing endometrial cancer but not ovarian cancer. The odds of women with a history of GDM or with the delivery of LGA infant developing endometrial cancer was 2.691 (95% CI: 1.548, 4.3635, p=0.0003), or 6.383 (95% CI: 2.812, 13.68, p<0.0001) respectively, compared to the controls. The odds ratio of women who did not use IUDs developing ovarian cancer was 1.606 (95% CI: 1.057, 2.434), compared to the controls. There was no association of age at first birth and developing endometrial or ovarian cancer.

**Conclusion:**

Our observational data suggested that GDM and delivery of an LGA infant are associated with an increased risk of endometrial cancer.

## Introduction

Endometrial and ovarian cancers are most common gynecological cancer worldwide (1). Despite the risk of cancer being greatly dependent on lifestyle factors, which may in many ways be different between multiparous and nulliparous women, strong evidence has previously suggested that women with multiparous have significantly decreased the incidence of endometrial and ovarian cancer (2–6). A recent study further reported that women with multiparous delay the time of developing endometrial cancer (7). Interestingly a recent study reported that women with multiparous have a similar overall risk of developing non-gynecological cancers compared to women with a lower parity (8). This study further confirmed a negative association of pregnancy with developing gynecological cancer.

The underlying mechanism of this long-term protective effect by multiparous has been suggested by the shift in hormonal balance (reduced levels of estrogen and elevated levels of progesterone) and lack of ovulation during pregnancy (5, 9, 10), although a recent study indicated that the shift in hormonal balance (increased levels of progesterone in third trimester) is not the sole explanation for the protective effect of pregnancy (11). Hormonal changes may affect malignant transformation such as causing DNA damage (12) and new evidence further shows that in general estrogen has an impact on carcinogenesis (13). Elevated progesterone levels during pregnancy could inhibit estrogen-driven endometrial cell proliferation and promote differentiation and apoptosis of endometrial cells (14, 15). It is also well-known that breasting feeding significantly reduces gynecological cancer development (16), which could be due to the reduced ovulation.

During pregnancy, in addition to other organs (such as ovary and uterine) the placenta also produces large amounts of estrogen and progesterone (17). Recent evidences have showed aberrant maternal serum concentration of estrogen and progesterone in complications of pregnancy, such as preeclampsia, intra-uterine growth restriction (IUGR) and preterm birth (18–22). Imbalance between estrogen and progesterone could also impair fetal maturation and growth in IUGR infants (22). It is well believed that placental dysfunction is associated with most of obstetrical complications and contributes to the aberrant production of estrogen and progesterone.

The association of complications of pregnancy and developing gynecological cancer has been recently investigated with limited data. Preeclampsia was reported to increase the risk of developing endometrial cancer (23) or ovarian cancer (24, 25), however other studies did not find the increased risk for developing endometrial cancer in women with a history of preeclampsia (24–27). Gestational diabetes mellitus (GDM) was reported to increase the risk for developing endometrial cancer (28) or ovarian cancer (29). While recent studies reported there is no association of GDM and the development of endometrial or ovarian cancer later in women’s life (30–32). These studies suggest that the association of complications of pregnancy with gynecological cancer is controversial. Therefore, in this observational case-control study, we investigated whether there is an association between complications of pregnancy and the incidence of endometrial and ovarian cancer.

## Methods

This study received approval by the Ethics Committee of The affiliated Wuxi Maternity and Child Health Care Hospital of Nanjing Medical University, China. Ethic Committees granted permission for data collection from hospital medical database.

### Study Cohort

A total of 189 women who were diagnosed with endometrial cancers and 119 women who were diagnosed with ovarian cancer from January 2013 to December 2016 from Wuxi Maternity and Child Health Hospital, Nanjing Medical University, China were included. In addition, during the study period, 343 women without gynecological cancer were randomly selected as a control group. All these selected women had more than one live birth. Age at diagnosis of endometrial or ovarian cancers, age at first birth, status of menopause, history of complications of pregnancy including preeclampsia, GDM and IUGR, Large for gestational age (LGA) were collected. In addition, parity, gravida, the length of breastfeeding and use of intra uterine devices (IUDs) were collected from the hospital’s electronic database.

Preeclampsia was defined as a maternal systolic blood pressure ≥140mmHg and/or diastolic blood pressure ≥ 90mmHg measured on two occasions separated by at least 6 hours, with or without proteinuria (>300 mg in a 24 hours period), and/or impaired liver function and/or lower platelet count, after 20 weeks of gestation in accordance with the guidelines of the International Society for the Study of Hypertension in Pregnancy (ISSHP) (33). GDM was defined as having any degree of glucose intolerance with onset or first recognition during pregnancy according to the World Health Organization (WHO) guidelines (http://www.who.int/iris/handle/10665/85975). LGA were defined as being of a birth weight at 90^th^ percentile of greater for gestational age (34). IUGR was defined by an estimated fetal weight below the 10^th^ percentile for its gestational age (35–37).

### Statistical Analysis

Age at diagnosis or age at first delivery was expressed as median and range. ANOVA or Chi-square test was performed to analyze the difference in clinical characteristics of study cohort and the association of complications of pregnancy and endometrial or ovarian cancer. The odds ratio (OR) 95% confidence intervals (CI) for the association of GDM or LGA and the risk of developing endometrial or ovarian cancer was performed using GraphPad Prism version 8.4, GraphPad Software, La Jolla, CA. p<0.05 was considered as the threshold for statistical significance.

## Results

### Clinical Characteristics of the Study Population

The general characteristics of study participants are summarized in **Table 1**. The median age was 53 (range 29-65) years in women with endometrial cancer, 50 (range 24-68) years in women with ovarian cancer and 43 (range 20-65) years in women in the control group. The age at first time of delivery was 25 (range 19-33) years in women with endometrial cancer, 24 (range 20-32) years in women with ovarian cancer, and 25 (range 18-36) years in controls. There was no difference in the age of first birth (p=0.248).

**Table 1:** Clinical characteristics of study population.

	Endometrial cancer (n=189)	Ovarian cancer (n=119)	Controls (n=343)	P value
Age at diagnosis (years, median/range)	53 (29-65)	50 (24-68)	43 (20-64)	<0.00001 (ANOVA)
Age at first birth (years, median/range)	25 (19-33)	24 (20-32)	25 (18-36)	0.248 (ANOVA)
Parity (number, %)				Chi square
P=1	128 (68%)	94 (79%)	280 (82%)	0.001
P≥2	61 (32%)	25 (21%)	63 (18%)	
Abortion (number, %)	N=136	N=79	N=260	Chi square
Once	76 (56%)	39 (49%)	134 (51%)	0.691
Twice	39 (29%)	22 (28%)	80 (31%)	
≥ Thrice	21 (15%)	18 (23%)	46 (18%)	

### GDM Was Associated With Developing Endometrial Cancer

Of 189 women diagnosed with endometrial cancer, there were 33 (17.5%) patients with a history of GDM, which was significantly higher than that in controls (7.3%) (**Table 2**). The odds ratio of women with a history of GDM developing endometrial cancer was 2.691 (95% CI: 1.548, 4.3635, p=0.0003), compared to the controls. In addition, there were 25 (13%) patients who delivered an LGA infant in their previous pregnancies, which was significantly higher than that in controls (2.3%) (**Table 2**). The odds ratio of women who delivered an LGA infant developing endometrial cancer was 6.383 (95% CI: 2.812, 13.68, p<0.0001), compared to the controls.

**Table 2:** The association of complications of pregnancy and endometrial cancer.

	Women with endometrial cancer (n=189)	Women without endometrial cancer (n=343)	P value (Chi-square test)
GDM (n, %)	33 (17.5%)	25 (7.3%)	0.0003
LGA (n, %)	25 (13%)	8 (2.3%)	<0.0001
Breastfeeding (n, %) *	93 (90%)	311 (93%)	0.447
Preeclampsia (n, %)	3 (1.6%)	6 (1.7%)	0.889
IUGR (n, %)	1 (0.5%)	5 (1.5%)	N/A
Using IUD (n, %)	100 (53%)	167 (49%)	0.621

*, data on 36 cases with endometrial cancer and 8 controls missed; N/A, not applicable.

In contrast, the portion of patients with a history of preeclampsia (1.6%) or with a history of IUGR (1.5%) was similar with controls (1.7% or 1.5%, respectively, **Table 2**). Due to the small sample size, we were not able to perform a statistical analysis. The portion of women diagnosed with endometrial cancer who used intrauterine devices (IUDs) (53%) was not statistically different to the controls (49%) (p=0.621). The portion of women diagnosed with endometrial cancer who had breastfeeding was also no difference to the controls (91% vs 93%, p=0.447).

### Complications of Pregnancy Was Not Associated With Developing Ovarian Cancer

Of 119 women diagnosed with ovarian cancer, there were 5 (4.2%) patients with a history of GDM, which was no different to the controls (2.3%) (**Table 3**, p=0.239). There were 5 (4.2%) patients who delivered an LGA infant in their previous pregnancies, which was also not statistically different to the controls (7.3%) (**Table 3**, p=0.288). In addition, the proportion of women with a history of preeclampsia (1.7%) or with a history of IUGR (0.8%) in women diagnosed with ovarian cancer was also no different to the controls (1.7% or 1.5%). Due to the small sample size, we were not able to perform a statistical analysis. The portion of women with ovarian cancer who had breastfeeding (90%) was also no difference to the controls (93%, p=0.315). However, the portion of women diagnosed with ovarian cancer who used intrauterine devices (IUDs) (39%) was significantly lower than the controls (49%) (**Table 3**, p=0.029). The odds ratio of women without using IUDs developing ovarian cancer was 1.606 (95% CI: 1.057, 2.435, p=0.029), compared to the controls.

**Table 3:** The association of complications of pregnancy and ovarian cancer.

	Women with ovarian cancer (n=119)	Women without ovarian cancer (n=343)	P value (Chi-square test)
GDM (n, %)	5 (4.2%)	25 (7.3%)	0.239
LGA (n, %)	5 (4.2%)	8 (2.3%)	0.288
Breastfeeding (n, %) *	93 (90%)	311 (93%) n=335	0.315
Preeclampsia (n, %)	2 (1.7%)	6 (1.7%)	N/A
IUGR (n, %)	1 (0.8%)	5 (1.5%)	N/A
Using IUD (n, %)	46 (39%)	167 (49%)#	0.029

*, data on 16 cases with ovarian cancer and 8 controls missed; #, data on 11 controls missed; N/A, not applicable due to small same size.

## Discussion

In this case-control study in Chinese population, we found that women with a history of GDM or with delivery of LGA infants, but not preeclampsia increased the risk of developing endometrial cancer. In contrast, women with a history of preeclampsia or GDM or with delivery of LGA infants or IUGR did not increase the risk of developing ovarian cancer. Use of IUDs only reduced the risk for developing ovarian cancer.

The pathophysiology of cancer associated to pregnancy is not fully understood. However, the negative association of parity and the incidence of endometrial and ovarian cancer can be explained by hormonal changes, immunological suppression and increased permeability and vascularization during pregnancy. Increased level of estrogen is one of the risk factors for developing endometrial or ovarian cancer through stimulating the growth of estrogen-dependent tissue (14, 15). During pregnancy, placenta also produces large amounts of hormones and placental dysfunction is now recognized as one of the pathogenesis of complications of pregnancy. The aberrant maternal serum concentration of estrogen and progesterone are reported in complications of pregnancy (18–22). Recent study also reported that the expression of estrogen receptor α (ERα) was increased in extravillous trophoblasts in GDM (38). This consequently suggest that complications of pregnancy may increase the risk of developing gynecological cancer. However, the findings recently reported in the literature are not consistent, due to the limited studies.

To date two recent studies investigated the association of GDM and the risk of developing endometrial cancer. Study led by Wartko reported that GDM increased the risk for developing endometrial cancer later in women’s life (28), while another study did not find this association (30). In our current study, we found that a history of GDM increased the risk of developing endometrial cancer with an odd ratio of 2.69, which is consistent with the Wartko’s study (28). It is well-known that GDM is associated with delivery of LGA infants (39, 40) and the association of delivery of LGA infants and the risk of developing endometrial cancer has not been well investigated. In our current study, we also found that delivery of LGA infants in previous pregnancy significantly increased the risk of developing endometrial cancer. The odd ratio in women with a delivery of LGA infant developing ovarian cancer was 6.4 (95% CI: 2.812, 13.68), compared to the controls.

To date, only four studies investigated the association of GDM and the risk of developing ovarian cancer. Study led by Fuchs (29) reported a significant positive association of GDM and endometrial cancer, and other two studies reported a positive association of GDM and endometrial cancer, but the association did not reach statistical difference (30, 32). While, a recent study did not find this association (31). In our current study, we also did not find a significant association between GDM and the risk of developing ovarian cancer.

Prior studies investigating the association of preeclampsia and risk of developing endometrial cancer or ovarian cancer are limited in the literature due to the relatively low prevalence of preeclampsia. Although a recent study reported an increased risk of developing endometrial cancer in women with a history of preeclampsia (23), four previous studies reported that women with a history of preeclampsia did not have an increased risk of developing endometrial cancer (24–27). In our current case-control study, we also did not find an association of preeclampsia and endometrial cancer. Due to the small sample size of preeclampsia, previous studies including our current study were not able to analyze whether the time of onset of preeclampsia is associated with the risk of developing endometrial cancer. It is now believed that placental dysfunction is more related to the pathogenesis of early onset preeclampsia while maternal response is likely involved in the pathogenesis of late onset preeclampsia (41). One study reported that early onset preeclampsia increased the risk of developing endometrial cancer (26). In addition, in our current study, we found that preeclampsia did not increase the risk of developing ovarian cancer, although previous two studies reported an association of preeclampsia and risk of developing ovarian cancer. This difference could be due the small sample size seen in our study. Preeclampsia and IUGR share very similar uteroplacental pathology and the association of women with IUGR and risk of developing gynecological cancer has not been well investigated yet. In our current study we found that IUGR did not increase the risk of developing either endometrial or ovarian cancer.

Across the board, the IUD is known to lower risk for many gynecological cancers, including endometrial (42) and ovarian cancer (43). In our current study, we found that the number of women with ovarian cancer who used IUDs was significantly lower than the controls, suggesting use of IUDs has a protective effect on developing ovarian cancer. This finding was consistent with previous study. However, we did not find the lower number of women diagnosed with endometrial cancer who used IUDs, compared to the controls. We do not know the exact reason causing this difference between previous study and our study. This could be due to the types of IUD and the duration of use between two studies.

Age at first birth has been suggested to be associated with the risk of developing endometrial cancer, but this finding is not consistent. A recent study with two population-based prospective cohort studies reported that age at first birth is unlikely to increase the risk of developing endometrial cancer (44). In our current study, we also found that there was no difference in age at first birth between women diagnosed with endometrial or ovarian cancer and the controls. However, due to the previous One-Child Policy in China, the majority of cases included in this study were uniparous (one birth). This may result in that the age at first birth was relatively younger.

We acknowledge that the sample size in our current study is relatively small and all the data were collected from a single tertiary hospital. In addition, the association of subtypes of endometrial or ovarian cancer and complications of pregnancy were not able to be further analyzed. Future study with a large sample size and multicenter care is required.

In this study, we found that GDM and delivery of LGA increased the risk of developing endometrial but not ovarian cancer. Although these two types of cancer are hormonal dependents, we do not know the exact reasons for our finding. Studies have reported that women with a history of GDM have a 7-fold higher risk of developing diabetes mellitus in women’s later life (45). Previous studies also found an association of endometrial cancer, but not ovarian cancer and diabetes mellitus (46–48), suggesting diabetes mellitus is a risk factor for developing endometrial cancer.

In this study, we reported the difference in the association of endometrial cancer with complicated pregnancy between GDM and preeclampsia. We do not know the exact reason for this finding, but this could be due to the difference in the pathogenesis of these two complications. A study reported an increased placental leptin expression in GDM, and leptin is one of the most probable candidates involved in the pathophysiology of GDM (49). While, levels of human chorionic gonadotrophin (hCG) and corticotrophin-releasing hormone (CRH) which are produced by the placenta are significantly increased in women with preeclampsia.

In conclusion, our current study reported that women with a history of GDM or with a delivery of LGA infant increased the risk of developing endometrial cancer but not ovarian cancer. In contrast, women with a history of preeclampsia or IUGR did not increase the risk of developing either endometrial or ovarian cancer. Age at first birth is unlikely to be associated with the risk of developing endometrial or ovarian cancer.

During pregnancy, your placenta makes hormones that cause glucose to build up in your blood. Usually, your pancreas can send out enough insulin to handle it. But if your body can’t make enough insulin or stops using insulin as it should, your blood sugar levels rise, and you get gestational diabetes.

## Data Availability Statement

The original contributions presented in the study are included in the article/supplementary material. Further inquiries can be directed to the corresponding author.

## Ethics Statement

The studies involving human participants were reviewed and approved by the Ethics Committee of The affiliated Wuxi Maternity and Child Health Care Hospital of Nanjing Medical University, China. Written informed consent for participation was not required for this study in accordance with the national legislation and the institutional requirements.

## Author Contributions

All authors contributed to the article and approved the submitted version. In addition to this, each author contributed to the following work: YL, XC, JS: collected the data reported in this work. XS: literature data collection. GC, MZ, QC: designed study and wrote the manuscript draft.

## Funding 

This study received the support from Outstanding Talent Project of Wuxi Health and Family Planning Commission of China (ZDRC023).

## Conflict of Interest

The authors declare that the research was conducted in the absence of any commercial or financial relationships that could be construed as a potential conflict of interest.
